# Effects of Exercise-Based Interventions on Functional Movement Capability in Untrained Populations: A Systematic Review and Meta-Analysis

**DOI:** 10.3390/ijerph19159353

**Published:** 2022-07-30

**Authors:** Jiafu Huang, Mengting Zhong, Jinghao Wang

**Affiliations:** School of Physical Education & Sports Science, South China Normal University, Guangzhou 510006, China; 2019020902@m.scnu.edu.cn (J.H.); 2021021125@m.scnu.edu.cn (M.Z.)

**Keywords:** exercise-based interventions, functional movement capability, untrained populations, functional movement screen

## Abstract

The purpose of this meta-analysis was to determine the effects of exercise-based interventions on functional movement capability in untrained populations and provide a reference for future intervention studies in this field. PubMed, Embase, Scopus, Web of Science, EBSCOhost, Cochrane Library, WanFang, and CNKI databases were systematically searched from inception until February 2022, for randomized or non-randomized controlled trials, addressing the effect of physical activity on functional movement capability in untrained populations. Two researchers independently conducted study selection, data extraction, and quality evaluation. Meta-analysis was performed using RveMan 5.3 and Stata 16.0 software. Twenty studies with 1596 participants were included in the meta-analysis. The results of meta-analysis demonstrated that exercise-based interventions were associated with improved asymmetry functional patterns (RR = 0.40; 95% CI [0.31, 0.50]; *p* < 0.00001), FMS composite score (MD = 3.01; 95% CI [2.44, 3.58]; *p* < 0.00001), deep squat (MD = 0.57; 95% CI [0.37, 0.77]; *p* < 0.00001), hurdle step (MD = 0.56; 95% CI [0.38, 0.75]; *p* < 0.00001), in-line lunge (MD = 0.54; 95% CI [0.43, 0.66]; *p* < 0.00001), shoulder mobility (MD = 0.37; 95% CI [0.15, 0.60]; *p* = 0.001), active straight leg raise (MD = 0.42; 95% CI [0.24, 0.60]; *p* < 0.00001), trunk stability push up (MD = 0.40; 95% CI [0.16, 0.63]; *p* = 0.001), and rotary stability (MD = 0.45; 95% CI [0.24, 0.67]; *p* < 0.0001). Exercise-based interventions were effective in improving functional movement capability in untrained populations. However, there is a need for high-quality, sufficiently powered RCTs to provide a more definitive conclusion.

## 1. Introduction

Functional movement capability is the ability to move effectively and competently in various fundamental movement patterns and motor skills, which is specifically characterized by the mobility, stability, coordination, and symmetry of fundamental movements in the human body [1,2]. Functional movement capability, as an important indicator to reflect the physical function of humans, represents an important building block for lifelong engagement and potentially injury-free engagement in sport activity [3]. Functional movement capacity is closely related to sports injuries. Studies have shown that nearly 80% of sports injuries are closely associated with the musculoskeletal system [4] and more than 70% of musculoskeletal injuries are caused by intrinsic risk factors [5]. Researchers believe that the main internal factor of musculoskeletal injuries is the functional movement dysfunction in the body, which is a neuromuscular symptom caused by dynamic postural instability [6,7]. The musculoskeletal screening test can identify and diagnose these dysfunctions of functional movement capacity, so that appropriate intervention programs can be developed to improve functional movement capability and prevent sports injury [8].

The functional movement screen (FMS) is used to evaluate fundamental movement patterns to identify potential risk factors, such as dysfunction, asymmetry, and pain, which is the most commonly used assessment tool for functional movement capability [9,10]. It comprises seven individual tests: deep squat, hurdle step, in-line lunge, shoulder mobility, active straight-leg raise, trunk stability push up, and rotary stability. Each test is scored on a scale of 0–3 to produce a total score out of 21. When the subjects obtain lower scores in FMS testing, they indicate less than optimal functional movement capability and the risk of injury during sports will increase [9,10]. A meta-analysis by Bonazza et al. reported that scoring ≤ 14 was associated with a small threefold increase in all-cause injury odds in an athlete, firefighting, and military population [11]. Another study indicated that higher FMS composite scores were associated with better dynamic balance in an active young male and female population, and participants who score > 14 on the FMS exhibited better dynamic balance than those with scores ≤ 14 [12]. Meanwhile, several studies found that FMS performance is significantly correlated with important health markers in the elderly, as well as gait stability and motor performance in young adults [13,14,15].

A lack of physical activity and exercise is one of the reasons for dysfunctional movement patterns. The stability and movement control components contained in exercise have a positive effect on functional movement capability [8]. Researchers have carried out a large number of related studies on the impact of exercise-based or physical activity interventions on functional movement capability recently. These studies mainly focused on athletes, firefighters, soldiers, and other professionally trained special occupational populations, and have reported relatively consistent results regarding exercise interventions, in that they can improve their functional movement capability and reduce the risk of sports injuries. For example, several studies have shown that exercise intervention programs can improve the FMS composite scores of athletes, firefighters, and military personnel, reduce asymmetry functional patterns, and reduce their risk of sports injuries [16,17,18,19,20]. A recent meta-analysis also indicated that functional correction training can improve athletes’ FMS composite scores and functional movement patterns and reduce their risk of sports injuries [21]. However, although researchers have also conducted some studies on the effect of exercise-based interventions on the functional movement capability of the untrained populations, there is still no consistent conclusion due to the influence of sample size, study design, and intervention program. For example, Shim et al. found that aerobic exercise can improve functional movements and FMS scores in elderly women, but the sample sizes in the experimental group (n = 9) and the control group (n = 10) in this study were smaller [22]. Yeon et al. concluded that Pilates can improve college students’ FMS scores and improve their functional movements, but the study adopted pre- and post-test design, lacking a control group [23]. In contrast, Wright et al. found that 4 weeks of fundamental movement training could not improve FMS performance in children, which may be due to short-term interventions [24]. Accordingly, it is urgent for researchers to seek an appropriate method to solve the current conflicting results.

Although there are differences in physical conditions and sports environment between untrained populations and professional groups, such as athletes, sports injuries are not just features of athletes. Identifying weaknesses in an untrained population and then trying to improve them could play an important role in lifelong physical activity and injury prevention. Systematic review is the highest level of evidence-based evidence by systematically collecting and screening relevant studies and strictly evaluating the quality of the included studies [25]. At present, there are no published meta-analyses or systematic reviews on the effect of exercise-based interventions on functional movement capability in untrained populations. Therefore, the purpose of the present meta-analysis was to investigate the effects of exercise-based interventions on functional movement capability in untrained populations and provide a reference for practical applications and clinical studies in this field in the future. We hypothesized that exercise-based interventions would improve the functional movement capability of untrained populations.

## 2. Materials and Methods

### 2.1. Design

This systematic review and meta-analysis were performed according to the Preferred Reporting Items for Systematic Reviews and Meta-Analysis statement (PRISMA) [26] and Cochrane Collaboration Handbook [27]. This review was prospectively registered with PROSPERO (CRD42022330725).

### 2.2. Search Strategy

An extensive literature search was conducted using eight electronic databases: PubMed, Embase, Scopus, Web of Science, EBSCOhost (including SportDiscus and Academic Search Premiere), Cochrane Library, WanFang, and China National Knowledge Infrastructure (CNKI) from inception to February 2022. The following combinations of terms were adapted for each database: (functional movement screen OR FMS OR functional movement screen*) AND (functional movement patterns OR movement quality OR injury risk OR injury prediction OR injury prevention OR injur*) AND (exercise OR physical activity OR functional training OR functional strength training OR movement training). The Chinese version of keywords “functional movement screen, movement quality, functional movement patterns and functional training” were also used. Any disagreements in the search process were resolved by discussion between two researchers (J.H. and M.Z.) and consulting the third researcher (J.W.). Additionally, reference lists of all included studies and any previous systematic reviews were also screened to identify additional eligible studies. The specific search syntax, such as PubMed, Embase, and Scopus, is available in the Supplementary Materials.

### 2.3. Selection Criteria

#### 2.3.1. Inclusion Criteria

The inclusion criteria were as follows: (1) English and Chinese language studies; (2) randomized controlled trials (RCTs) or non-randomized controlled trials (non-RCTs); (3) participants were untrained populations who did not engage in any systematic training (except for special occupation groups such as professional athletes, firefighters, military personnel, and police), without restrictions on gender, age and region; (4) intervention focusing on a preventive training program or sport, including a set of exercise-based/physical activity interventions aimed at improving stability, mobility, coordination or symmetry; (5) outcome measures included FMS composite score, FMS individual score and/or FMS asymmetry after intervention in the experimental group and control group.

#### 2.3.2. Exclusion Criteria

The studies were excluded if: (1) they were meeting abstracts, case reports, conference proceedings, or reviews; (2) duplicated studies; (3) the topic irrelevant to this review; (4) insufficient data or lack of outcome indicators; (5) participants were athletes; and (6) they were cross-sectional or retrospective studies.

### 2.4. Study Selection and Data Extraction

The two researchers (J.H. and M.Z.) independently screened the title, abstract, and full texts according to the predetermined criteria. Meanwhile, the following data were independently extracted by two researchers (J.H. and M.Z.): publication details (first author and publication date), participant characteristics (mean age/age range, and sample size); exercise interventions (type, period, frequency and time); outcomes (FMS composite score, FMS individual score and/or the incidence of FMS asymmetry) and study design. Discrepancies were resolved by discussion or the third researcher (J.W.) was consulted.

### 2.5. Risk of Bias

The quality of the included studies was assessed by two independent researchers (J.H. and M.Z.) and disagreements were resolved by consensus. The quality of RCT studies was evaluated by PEDro scale, an internationally recognized and widely used evaluation tool, which added two indicators on the basis of Delphi scale [28]. This scale includes 11 items as follows: eligibility criteria, randomized allocation, concealed allocation, similar baseline, blinding of participants, blinding of therapists, blinding of assessors, less than 15% dropouts, intention-to-treat analysis, between-group comparison, and point measure and measures of variability. The first item is not included to calculate the total PEDro score, so the maximum score was 10 points. Each item was only scored as ‘yes’ or ‘no’. RCT studies were classified as having excellent (9–10), good (6–8), fair (4–5), or poor (<4) quality, respectively. The MINORS scale was used to evaluate the quality of non-RCT studies [29]. The MINORS scale contains 12 items, the first 8 being specifically for non-comparative studies. Each item is scored on a scale of 0–2 for a total score of 24 points, 0 indicating that it is not reported, 1 indicating that it is reported but insufficient, and 2 indicating that it is reported sufficient. Non-RCT studies were classified as low quality (0–8), medium quality (9–16), or high quality (17–24), respectively. Studies were excluded if they scored less than 12.

### 2.6. Data Analysis and Synthesis

The RevMan 5.3 software (Cochrane Collaboration, Oxford, UK) was used to perform meta-analysis: effect size combination, heterogeneity test, sensitivity analysis, and subgroup analysis. The Stata 16.0 software (StataCorp, College Station, TX, USA) was used to carry out funnel plot and Egger’s test to detect potential publication bias [30]. For dichotomous variables, the risk ratio (RR) was used to combine the asymmetry functional patterns. For continuous variables, the weighted mean difference (WMD) was used to combine FMS composite score and FMS individual score. The heterogeneity of the results across studies was evaluated using the I^2^ statistical. When I^2^ < 50%, the fixed effect model was adopted to perform meta-analysis; otherwise, the random effect model was used. The heterogeneity I^2^ statistic was divided into three grades: small (25%), moderate (50%), and high (75%). If the heterogeneity was too large or the effect sizes could not be combined, which is not suitable for meta-analysis, qualitative synthesis analysis was performed. Finally, the precision of the effect sizes was described using 95% confidence intervals (CIs) and the significant difference was *p* < 0.05.

## 3. Results

### 3.1. Study Selection

**Figure 1:**
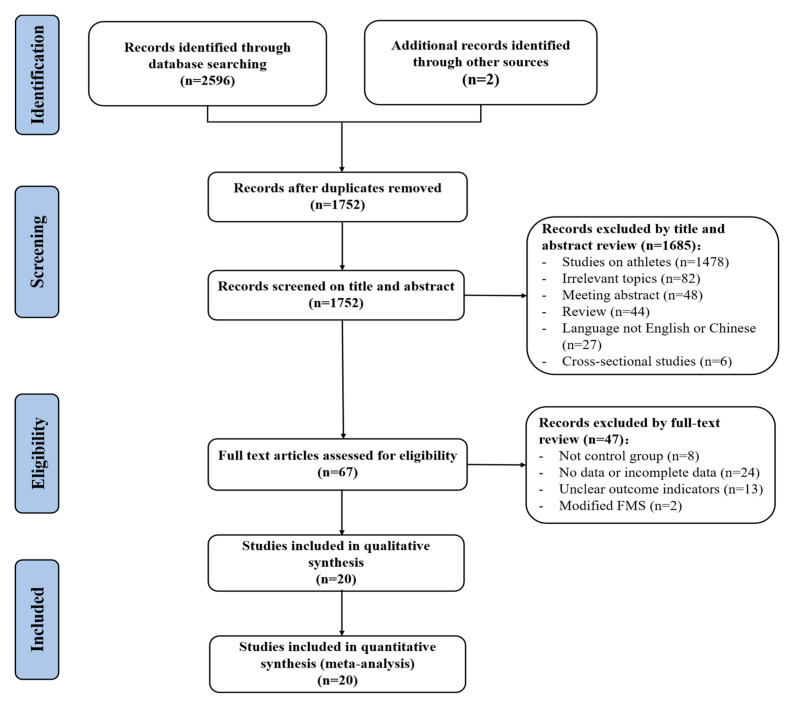
Flow diagram of the study selection.

In total, 2598 studies were retrieved from eight databases and other resources. These studies were imported into EndNote X9 (Thomson Research Soft, Stanford, CA, USA), and duplicates (n = 864) were removed. Of the remaining 1752 studies, 1685 were eliminated after screening the title and abstract. The remaining 67 studies were further screened by reading full texts and 47 studies were excluded. Finally, 20 studies provided sufficient information to be included in the meta-analysis. The detailed searching and screening process of the study is shown in Figure 1.

### 3.2. Study Characteristics

In total, 20 studies were selected in this study, including 15 RCTs [8,31,32,33,34,35,36,37,38,39,40,41,42,43,44] and 5 non-RCTs [45,46,47,48,49]. All were published between 2016 and 2022 as peer-reviewed articles or dissertations. The study included a total of 1596 participants; 834 were included in the experimental group and 729 in the control group. The average sample size of each study was 80, ranging from 24 to 233. Participants involved healthy children, adolescents, and middle-aged and elderly people, and their ages ranged from 8 to 65.42 years. The exercise-based interventions can be divided into personalized training programs (functional training, functional strength training, core stability training, etc.) and specific sports (Tai Chi, Yoga, Pilates, Health Qigong, etc.). The intervention period ranged from 6 to 24 weeks, and 12 weeks was the most used. The intervention frequency ranged from 1 to 6 times per week and 3 times per week was the most adopted. The intervention time varied from 20 to 90 min and 60 min was the most used. It is worth mentioning that the purpose of a study is to examine the acute effects of interventions, so the intervention period and frequency are not provided [36]. The outcome measures included the incidence of asymmetry functional patterns, FMS composite scores, and FMS individual scores. In addition, two studies used a three-arm experiment design and one study consisted of two experiments, so two sets of data for these studies were extracted for meta-analysis [41,42,43]. The basic characteristics of the included studies are shown in Table 1.

### 3.3. Risk-of-Bias Assessment

In this review, the PEDro scale and MINORS scale were used to evaluate the quality of 15 RCT studies and 5 non-RCT studies, respectively. The score of 15 RCTs was between 6 and 8 points, with an average score of 6.33 points, indicating the quality of RCTs was good. Two studies were randomly assigned with concealed allocation [36,44]; three studies were blinded, one of which was a double-blind trial [32], and two were a single-blind trial [36,42]. The scores of the five non-RCT studies were between 15 and 18 points, with an average score of 16 points, indicating that the non-RCTs were medium quality. One of the studies was high quality [48] and the others were medium quality [46,47,48,49]. None of the five non-RCTs reported blinding, follow-up time, and calculation of sample size, and only one study reported the loss to follow-up rate [48] (Table 2 and Table 3).

### 3.4. Meta-Analysis

#### 3.4.1. Asymmetry Functional Patterns

**Figure 2:**
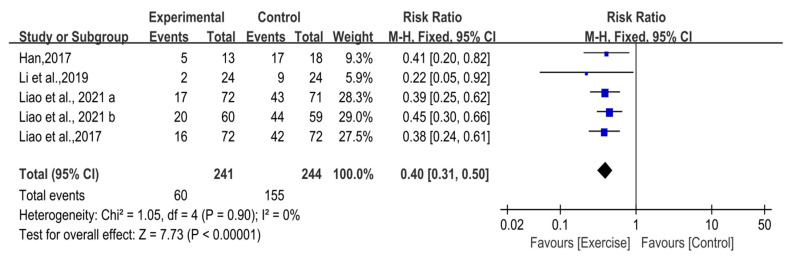
Forest plot of the untrained populations’ asymmetry functional patterns [35,43,44,45,49].

In total, 20 studies were included in this study, of which 4 studies (n = 485) provided sufficient data for meta-analysis of the incidence of asymmetry functional patterns of exercise-based interventions in untrained populations. A heterogeneity test showed no significant heterogeneity in the included studies (I^2^ = 0%; *p* > 0.1), so the fixed-effect model was adopted to combine the effect sizes. The result of meta-analysis showed that there was a significant difference between the experimental group and control group (RR = 0.40; 95% CI [0.31, 0.50]; Z = 7.73; *p* < 0.00001), suggesting that exercise-based interventions can significantly reduce the incidence of asymmetry functional patterns in untrained populations (Figure 2).

#### 3.4.2. FMS Composite Scores

**Figure 3:**
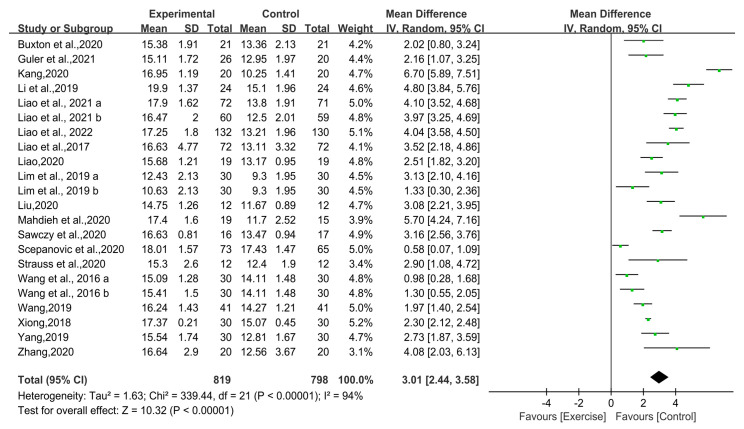
Forest plot of the untrained populations’ FMS composite scores [8,31,32,33,34,35,36,37,38,39,40,41,42,43,44,45,46,47,48].

Nineteen studies (n = 1505) compared the effect of FMS composite scores between the exercise group and the control group among the 20 included studies. The heterogeneity test showed high heterogeneity in the 19 studies (I^2^ = 94%; *p* < 0.00001), so the random-effect model was used to integrate the effect sizes. The result of the meta-analysis indicated a significant improvement in the exercise group compared with the control group (MD = 3.01; 95% CI [2.44, 3.58]; Z = 10.32; *p* < 0.00001), suggesting that exercise-based interventions can improve the FMS composite scores of untrained populations (Figure 3).

#### 3.4.3. FMS Individual Scores

**Figure 4:**
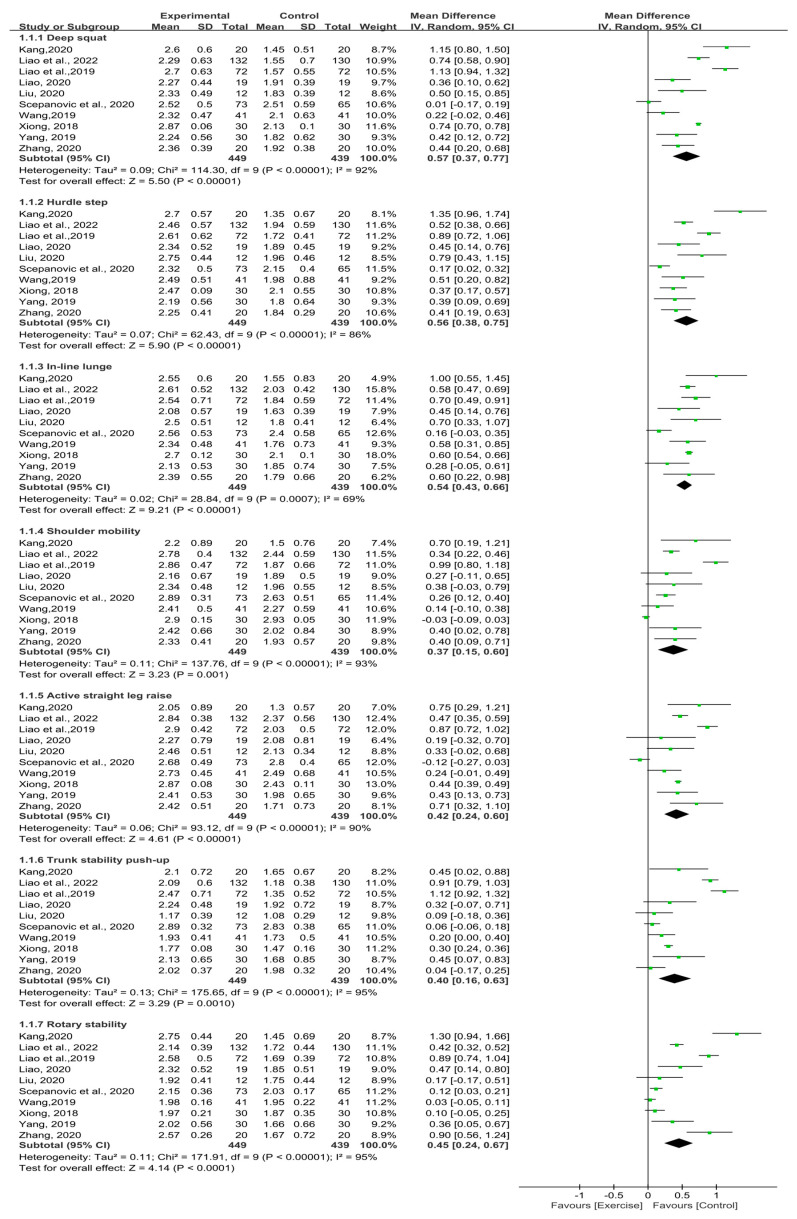
Forest plot of the untrained populations’ FMS individual scores [31,32,33,34,35,40,44,46,47,48].

Of the 20 included studies, 10 studies (n = 888) provided adequate information of seven individual FMS scores after exercise-based interventions in untrained populations, including deep squat, hurdle step, in-line lunge, shoulder mobility, active straight-leg raise, trunk stability push up, and rotary stability. We adopted meta-analytic methods to individually synthesize the study findings of each outcome. As there was high heterogeneity (I^2^ > 50%), the random-effect model was used to combine the effect sizes. The overall results showed significant benefit in favor of exercise-based interventions on improving deep squat (MD = 0.57; 95% CI [0.37, 0.77]; Z = 5.50; *p* < 0.00001), hurdle step (MD = 0.56; 95% CI [0.38, 0.75]; Z = 5.90; *p* < 0.00001), in-line lunge (MD = 0.54; 95% CI [0.43, 0.66]; Z = 9.21; *p* < 0.00001), shoulder mobility (MD = 0.37; 95% CI [0.15, 0.60]; Z = 3.23; *p* = 0.001), active straight-leg raise (MD = 0.42; 95% CI [0.24, 0.60]; Z = 4.61; *p* < 0.00001), trunk stability push up (MD = 0.40; 95% CI [0.16, 0.63]; Z = 3.29; *p* = 0.001), and rotary stability (MD = 0.45; 95% CI [0.24, 0.67]; Z = 4.14; *p* < 0.0001) (Figure 4).

### 3.5. Subgroup Analysis

In order to explore the source factors of heterogeneity, this review conducted a subgroup analysis of FMS composite scores. The effect of exercise-based interventions on the FMS composite scores of the untrained populations may be affected by different ages, intervention types, intervention time, intervention frequency, and intervention period. Therefore, we conducted a subgroup analysis of FMS composite score based on the above factors. They were divided into different subgroups as follows: (1) age: under 18 years old, 18–30 years old, and above 50 years old; (2) intervention: specific exercises and functional training programs; (3) time: Under 60 min, 60 min, and more than 60 min; (4) frequency: under three times a week, three times a week, and more than three times a week; and (5) period: 6 weeks, 8 weeks, and 12 weeks. Studies with unclear age and intervention characteristics were excluded [36,46]. The results of subgroup analysis showed that the heterogeneity of age, intervention type, intervention time, intervention frequency, and intervention period decreased, which indicates that these factors may be the source of heterogeneity in FMS composite scores, and intervention time is the most likely source of heterogeneity. Additionally, the result of subgroup analysis also showed that 6-week exercise-based interventions could not improve FMS composite scores in untrained populations (*p* > 0.05) (Table 4).

### 3.6. Sensitivity Analysis

Sensitivity analysis is a method to test the stability of the obtained results by assuming conditions. In this review, we conducted a sensitivity analysis of the meta-analysis results for the FMS composite scores and FMS individual scores with high levels of heterogeneity and combined the effect sizes of the remaining studies by eliminating individual studies one by one. In this review, the combined effect size of exercise-based interventions on FMS composite scores in untrained populations was MD = 3.01; 95% CI [2.44, 3.58]; *p* < 0.00001; I^2^ = 94%. When the studies were eliminated one by one, the effect size was MD = 2.81–3.13; I^2^ = 92%–94%; *p* < 0.00001. The effect sizes of exercise-based interventions of FMS individual scores in untrained populations were as follows: (1) The effect size of deep squat was MD = 0.57; 95% CI [0.37, 0.77]; *p* < 0.00001; I^2^ = 92%. When the studies were eliminated one by one, the effect size was MD = 0.50–0.64; I^2^ = 86%–93%; *p* < 0.0001. (2) The combined effect size of hurdle step was MD = 0.56; 95% CI [0.38, 0.75]; *p* < 0.00001; I^2^ = 86%. When the studies were eliminated one by one, the effect size was MD = 0.49–0.61; I^2^ = 79%–87%; *p* < 0.00001. (3) The combined effect size of in-line lunge was MD = 0.54; 95% CI [0.43, 0.66]; *p* < 0.00001; I^2^ = 69%. When one study was removed [48], the effect size was MD = 0.60; 95% CI [0.54, 0.66]; *p* < 0.00001; I^2^ = 10%. (4) the combined effect size of shoulder mobility was MD = 0.37; 95% CI [0.15, 0.60]; *p* = 0.001; I^2^ = 93%. When the studies were removed one by one, the effect size was MD = 0.28–0.43; I^2^ = 84%–94%; *p* < 0.05. (5) The combined effect size of active straight-leg raise was MD = 0.42; 95% CI [0.24, 0.60]; *p* < 0.00001; I^2^ = 90%. When two studies were eliminated [35,48], the effect size was MD = 0.44; 95% CI [0.38, 0.50]; *p* < 0.00001; I^2^ = 8%. (6) The effect size of trunk stability push-up was MD = 0.40; 95% CI [0.16, 0.63]; *p* = 0.001; I^2^ = 95%. When studies were eliminated one by one, the effect size of MD = 0.31–0.44; I^2^ = 91%–95%; *p* < 0.05. (7) The combined effect size of rotatory stability was MD = 0.45; 95% CI [0.24, 0.67]; *p* < 0.0001; I^2^ = 95%. When studies were eliminated one by one, the effect size of MD = 0.37–0.51; I^2^ = 92%–95%; *p* < 0.05.

### 3.7. Publication Bias

**Figure 5:**
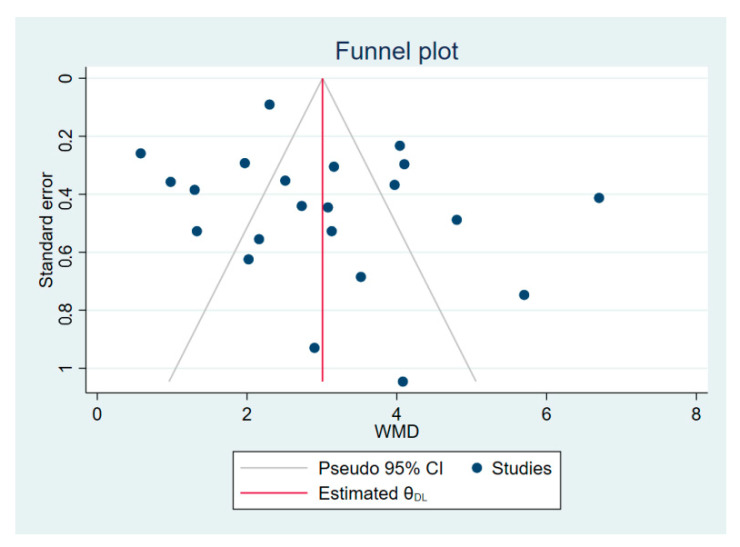
Funnel plot of publication bias for FMS composite scores.

The funnel plot analyses is performed to examine potential publication bias if the meta-analysis included more than 10 studies [50]. The meta-analysis of FMS composite scores showed no significant publication bias, as evidenced by visual inspection of the funnel plot and Egger’s regression test (*p* = 0.30 > 0.05) (Figure 5).

## 4. Discussion

This present systematic review and meta-analysis aimed to examine the effects of exercise-based interventions on functional movement capability in untrained populations, in order to provide reference for intervention research in this field. In total, 20 studies with 1596 participants (i.e., children, adolescents, middle-aged, and elderly) were included in this meta-analysis. The types of exercise intervention included functional training programs and specific sports (Tai Chi, Yoga, Health Qigong, etc.). Despite the different exercise-based interventions and participant characteristics, the findings of this review indicated that exercise-based interventions have a positive effect on functional movement capability in untrained populations.

### 4.1. Effect of Exercise-Based Interventions on Functional Movement Capability in Untrained Populations

The results of the meta-analysis showed that exercise-based or physical activity interventions can effectively improve the functional movement capability in untrained populations, which is manifested in improvements in the untrained population’s FMS composite scores and FMS individual scores and a reduction in the incidence of asymmetry movement patterns. A recent meta-analysis of exercise interventions on athletes’ functional movement capability also found that functional correction training can improve FMS composite scores and asymmetry movement patterns and reduce the risk of sports injuries [21]. There are five symmetrical movements in FMS that need to be tested on both sides of the body. The asymmetry functional patterns refer to at least one FMS test difference between the left and right sides of the body during FMS testing, and the scores obtained are inconsistent. This meta-analysis showed that exercise-based interventions can reduce the incidence of asymmetry functional patterns among untrained populations, which is consistent with the results of previous studies. Two studies found that Tai Chi and Yi Jinjing can effectively improve asymmetry functional patterns in college students [45,49]. Liao et al. also reported that functional strength training significantly improved the asymmetry functional patterns of 12–13-year-old girls [35]. The effect of exercise-based interventions on asymmetry functional patterns may be related to the characteristics of exercise. For example, Tai Chi, Yi Jinjing, Baduanjin, and other sports belong to bilateral sports [51]. The movement characteristics and arrangement form of these sports can reflect symmetry, and long-term exercise is conducive to the coordinated development of the practitioners’ bilateral functions.

The results of this meta-analysis demonstrated that exercise-based interventions also significantly improved the FMS composite scores of untrained populations. FMS composite scores is an important indicator of individual functional movement capability, with higher scores indicating better movement capability [52]. Laurent et al. also confirmed the conclusion that exercise-based interventions can improve FMS composite scores in an RCT study on the effect of a suspension-trainer-based movement program on children’s functional movements [53]. In addition, in the research exploring the relationship between exercise-based interventions and FMS composite scores, researchers found that an individual’s physical activity level is positively correlated with its FMS composite scores, which also confirmed the conclusion that exercise-based or physical activity interventions had significant effects on functional movement capability in this study [54,55,56,57].

For FMS individual scores, the results of this meta-analysis were consistent with previous studies [35,40], showing that exercise-based interventions significantly improved the FMS individual scores in untrained populations. Early research reported that 12 weeks of elastic band resistance training can improve the individual FMS scores of sedentary office workers [40]. Liao et al. found that functional strength training has a similar effect in improving FMS individual scores and movement quality in untrained healthy girls, aged 12–13 years [35]. Furthermore, some studies believe that more attention should be paid to the score of each task instead of the FMS composite scores when interpreting the FMS scores [58]. Several studies have also shown that individual FMS scores can better reflect individual performance and predict the risk of injury than FMS total scores [59,60,61]. At present, however, many studies mainly focus on the FMS composite score, and individual FMS score is easy for researchers to ignore. Therefore, more attention should be paid to the role of FMS individual scores in future studies on the effect of exercise interventions on functional movement capability.

### 4.2. Subgroup Analysis of FMS Composite Scores

Subgroup analysis showed that exercise interventions have a positive effect on the total FMS scores in untrained populations at different ages, but the effect was more significant for the middle-aged and elderly population over 50 years old, which may be affected by age factors. Studies found that FMS score is correlated with the age of middle-aged and elderly people, and FMS score decreases with age [56,62]. Therefore, compared with children and adolescents, the FMS scores of middle-aged and elderly people improved more significantly after exercise-based interventions under the same conditions. Subgroup analysis of exercise interventions showed that both specific sports, such as Tai Chi, Yoga, and functional training programs, could improve the FMS composite scores in untrained populations. Different from rugby, volleyball, fighting, and other competitive sports that over-emphasize the practice of sport-specific skills and ignore the development of whole-body functional movements, it is easy to cause poor functional movement capability in athletes and increase the risk of sports injuries. Functional training programs and specific sports include various movements, such as step, squat, and lunge, mainly focusing on the practice of movement forms, which are more conducive to the overall development of individual functional movement capability. This study is unable to draw a conclusion about which type of exercise-based or physical activity intervention is more effective, which is a topic worthy of attention in future studies. However, according to the characteristics of exercise intervention, we can provide some suggestions for people to choose exercise-based or physical activity intervention. For example, compared with other types of exercise intervention, mind–body exercises (i.e., Tai Chi, Yoga, Health Qigong, and Pilates) are low impact, moderate intensity, and emphasize trinity of mind, body, and breathing, which is more suitable for middle-aged and elderly people to practice [63,64,65]. The functional training program is mainly composed of different functional movements or instrument movements, and its exercise intensity and difficulty are relatively high, which may be more in line with the needs of young people [66]. For the period, frequency, and time of exercise-based interventions, subgroup analysis showed that exercise-based interventions occurring more than three times per week and 60 min per session for 12 weeks had a more significant improvement effect on the FMS composite score in untrained populations. This is not only basically consistent with the exercise prescription guidelines recommended by the American College of Sports Medicine for healthy people [67], but also confirms the conclusion reported in previous studies that 4-week short-term exercise-based interventions cannot improve FMS performance [24].

### 4.3. Sensitivity Analysis

In the present review, when the literature was removed one by one, the results of sensitivity analysis for FMS composite scores, deep squat, rotary stability, hurdle step, shoulder mobility, and trunk stability push-up showed that there was still high heterogeneity, and the results of effect size remained significant. This suggests that exercise interventions can improve the FMS composite scores in untrained populations, as well as the scores of deep squat, rotary stability, hurdle step, shoulder mobility, and trunk stability push-up. The sensitivity analysis results of in-line lunge and active straight-leg raise indicated that the heterogeneity is significantly reduced when the literature was eliminated one by one, but the effect size did not change significantly and there is no significant impact on the results. In summary, this indicates that the combined effect size results of the meta-analysis outcomes are relatively robust and reliable.

### 4.4. Strengths and Limitations

To the authors’ knowledge, this is the first review to investigate the effect of exercise-based interventions on functional movement capability in untrained populations. The quality of the literature included in this study is high, there is no publication bias among the studies, and the sensitivity analysis results are relatively robust and reliable. However, this review also has the following limitations: First, only English and Chinese literature was included in this review, which may cause language bias. Second, due to the limitation of literature quantity, both RCT and non-RCT studies were included in this review, and only two studies displayed concealed allocation plus three studies that reported blinding. Third, the meta-analysis results of FMS composite scores have high levels of heterogeneity. Although subgroup analysis was conducted and the possible source factors of heterogeneity were explored, heterogeneity still could not be eliminated, which may also have a certain impact on the results.

## 5. Conclusions

This meta-analysis demonstrated that exercise-based interventions have a positive effect on functional movement capacity in untrained populations. However, due to the lack of adequate high-quality RCTs, the findings of this review should be interpreted carefully. Therefore, more high-quality RCTs of exercise interventions on the functional movement capacity in untrained populations should be conducted in future research, and the impact of different interventions on the functional movement capacity of the untrained populations at different ages should be considered, so as to provide more substantial evidence for clinical research and practical applications in this field.

## Figures and Tables

**Table 1:** Characteristics of included studies (n = 20).

Authors, Year	Study Design	Participants (n)	Age (Years) (±SD)	Experimental Group	Control Group	Duration/Frequency /Period	Outcomes (Measures)
Buxton et al. (2020) [39]	RCT	College students (42) (EG: 21; CG: 21)	EG (19.38 ± 1.36); CG (20.14 ± 2.63)	Quadrupedal movement training	Waiting list	60 min, 2 times per week, 8 weeks	FMS composite score
Guler et al. (2021) [38]	RCT	Middle-aged adults (46) (EG: 26; CG: 20)	EG (51.55 ± 3.73); CG (52.85 ± 4.01)	Functional strength training	Traditional strength training	60 min, 3 times per week, 8 weeks	FMS composite score
Han (2017) [49]	Non-RCT	College students (31) (EG: 13; CG: 18)	NR	Yi Jinjing	Routine exercise	90 min, 3 times per week, 12 weeks	Asymmetry functional patterns
Kang (2020) [31]	RCT	Children (40) (EG: 20; CG: 20)	EG (9.45 ± 1.36); CG (9.50 ± 1.15)	Functional training	Routine exercise	90 min, once a week, 14 weeks	FMS composite score; FMS individual score
Li et al. (2019) [45]	Non-RCT	Male college students (48) (EG: 24; CG: 24)	18.88 ± 0.68	Simplified 24-form Tai Chi	Waiting list	20 min, 2 times per week, 8 weeks	FMS composite score; Asymmetry functional patterns
Liao et al. (2019) [35]	RCT	Girls (144) (EG: 72; CG: 72)	12.47 ± 0.57	Functional strength training	Traditional strength training	45 min, 3 times per week, 12 weeks	FMS composite score; FMS individual score; Asymmetry functional patterns
Liao (2020) [40]	RCT	Office sedentary people (38) (EG: 19; CG: 19)	EG (28.15 ± 1.9); CG (27.10 ± 2.1)	Elastic band resistance training	Waiting list	50–60 min, 3 times per week, 12 weeks	FMS composite score; FMS individual score
Liao et al. (2021) [43]	RCT	Adolescents (266) (EG1: 72; CG1: 72; EG2: 61; CG2: 61)	13–16	Functional strength training	Physical education	45 min, 3 times per week, 12 weeks	FMS composite score; Asymmetry functional patterns
Liao et al. (2022) [44]	RCT	Adolescents (266) (EG: 133; CG: 133)	EG (14.37 ± 0.55); CG (14.03 ± 0.59)	Functional strength training	Physical education	45 min, 3 times per week, 12 weeks	FMS composite score; FMS individual score
Lim et al. (2019) [41]	RCT	Adults (90) (EG1: 30; EG2: 30; CG: 30)	30–40	EG1:Pilates EG2:Yoga	Waiting list	60 min, 3 times per week, 8 weeks	FMS composite score
Liu (2020) [33]	RCT	Elderly adults (24) (EG: 12; CG: 12)	EG (65.25 ± 3.93); CG (65.42 ± 3.94)	Wu Qinxi	Waiting list	60 min, 6 times per week, 12 weeks	FMS composite score; FMS individual score
Mahdieh et al. (2020) [8]	RCT	Female students (34) (EG: 19; CG: 15)	EG (18.8 ± 0.68); CG (18.9 ± 0.91)	Dynamic neuromuscular stabilization training	Routine exercise	50 min, 3 times per week, 6 weeks	FMS composite score
Sawczy et al. (2020) [37]	RCT	College students (33) (EG: 16; CG: 17)	21.6 ± 1.3	Functional strength training	Routine exercise	60 min, 4 times per week (1–6 wk)/2 times per week (7–12 wk), 12 weeks	FMS composite score
Scepanovic et al.(2020) [48]	Non-RCT	Male college students (138) (EG: 73; CG: 65)	EG (20 ± 0.5); CG (20 ± 0.7)	Core stabilization training	Routine exercise	30 min, 3 times per week, 6 weeks	FMS composite score; FMS individual score
Strauss et al. (2020) [36]	RCT	Active young population (24) (EG: 12; CG: 12)	EG (25.7 ± 4.70); CG (27.4 ± 5.50)	Total Motion Release	Waiting list	2 sets of 15 repetitions	FMS composite score
Wang et al. (2016) [42]	RCT	Older adults (90) (EG1: 30; EG2: 30; CG: 30)	EG1 (65.2 ± 5.0); EG2 (65.3 ± 4.3); CG (65.3 ± 4.4)	EG1:Traditional Tai Chi EG2:Simplified Tai Chi	Routine activity	60 min, 4 times per week, 12 weeks	FMS composite score
Wang (2019) [32]	RCT	Female college students (82) (EG: 41; CG: 41)	NR	Modified yoga	Regular yoga	90 min, once a week, 12 weeks	FMS composite score; FMS individual score
Xiong (2018) [34]	RCT	Middle-aged women (60) (EG: 30; CG: 30)	50 ± 3.21	Yoga	Waiting list	60 min, 3 times per week, 12 weeks	FMS composite score; FMS individual score
Yang (2019) [47]	Non-RCT	Primary school students (60) (EG: 30; CG: 30)	8–10	Functional training	Waiting list	45 min, 2 times per week, 12 weeks	FMS composite score; FMS individual score
Zhang (2020) [46]	Non-RCT	College students (40) (EG: 20; CG: 20)	NR	Dao Yin	Routine exercise	90 min, 5 times per week, 24 weeks	FMS composite score; FMS individual score

Note: RCT = randomized controlled trial; non-RCT: non-randomized controlled trial; EG = experimental group; CG = control group; NR = not reported.

**Table 2:** Quality assessment of the included RCT studies with PEDro criteria (n = 15).

Authors, Year	Item 1	Item 2	Item 3	Item 4	Item 5	Item 6	Item 7	Item 8	Item 9	Item 10	Item 11	Total
Buxton et al. (2020) [39]	Y	Y	N	Y	N	N	N	Y	Y	Y	Y	6/10
Guler et al. (2021) [38]	Y	Y	N	Y	N	N	N	Y	Y	Y	Y	6/10
Kang (2020) [31]	Y	Y	N	Y	N	N	N	Y	Y	Y	Y	6/10
Liao et al. (2019) [35]	Y	Y	N	Y	N	N	N	Y	Y	Y	Y	6/10
Liao (2020) [40]	Y	Y	N	Y	N	N	N	Y	Y	Y	Y	6/10
Liao et al. (2021) [43]	Y	Y	N	Y	N	N	N	Y	Y	Y	Y	6/10
Liao et al. (2022) [44]	Y	Y	Y	Y	N	N	N	Y	Y	Y	Y	7/10
Lim et al. (2019) [41]	Y	Y	N	Y	N	N	N	Y	Y	Y	Y	6/10
Liu (2020) [33]	Y	Y	N	Y	N	N	N	Y	Y	Y	Y	6/10
Mahdieh et al. (2020) [8]	Y	Y	N	Y	N	N	N	Y	Y	Y	Y	5/10
Sawczy et al. (2020) [37]	Y	Y	N	Y	N	N	N	Y	Y	Y	Y	6/10
Strauss et al. (2020) [36]	Y	Y	Y	Y	N	Y	N	Y	Y	Y	Y	8/10
Wang et al. (2016) [42]	Y	Y	N	Y	N	N	Y	Y	Y	Y	Y	7/10
Wang (2019) [32]	N	Y	N	Y	Y	Y	N	Y	Y	Y	Y	8/10
Xiong (2018) [34]	Y	Y	N	Y	N	N	N	Y	Y	Y	Y	6/10

Note: N = does not meet the criteria; Y = meet the criteria; Item 1 = Eligibility criteria; Item 2 = Random allocation; Item 3 = Concealed allocation; Item 4 = Similar at baseline; Item 5 = Subjects blinded; Item 6 = Therapists blinded; Item 7 = Assessors blinded; Item 8 = <15% dropouts; Item 9 = Intention-to-treat analysis; Item 10 = Between-group comparisons; Item 11 = Point measures and variability data.

**Table 3:** Quality assessment of the included non-RCT studies with MINORS (n = 5).

Authors, Year	Item 1	Item 2	Item 3	Item 4	Item 5	Item 6	Item 7	Item 8	Item 9	Item 10	Item 11	Item 12	Total
Han (2017) [49]	2	1	2	2	0	0	0	0	2	2	2	2	15/24
Li et al. (2019) [45]	2	2	2	2	0	0	0	0	2	2	2	2	16/24
Scepanovic et al. (2020) [48]	2	2	2	2	0	0	2	0	2	2	2	2	18/24
Yang (2019) [47]	2	2	2	2	0	0	0	0	2	2	2	2	16/24
Zhang (2020) [46]	1	2	2	2	0	0	0	0	2	2	2	2	15/24

Note: 0 (not reported); 1 (reported but inadequate); 2 (reported and adequate). Item 1 = A clearly stated aim; Item 2 = Inclusion of consecutive patients; Item 3 = Prospective collection of data; Item 4 = Endpoints appropriate to the aim of the study; Item 5 = Unbiased assessment of the study endpoint; Item 6 = Follow-up period appropriate to the aim of the study; Item 7 = Loss to follow-up less than 5%; Item 8 = Prospective calculation of the study size; Item 9 = An adequate control group; Item 10 = Contemporary groups; Item 11 = Baseline equivalence of groups; Item 12 = Adequate statistical analyses.

**Table 4:** Subgroup analysis of the untrained populations’ FMS composite scores.

Group	Subgroup	N	MD	95% CI	*p*	*I* ^2^
Age (year)	Under 18	6	4.20	3.27, 5.12	<0.00001	90%
	18–30	9	2.99	1.97, 4.01	<0.00001	92%
	More than 50	5	1.95	1.28, 2.62	<0.00001	82%
Intervention	Specific sports	9	2.42	1.80, 3.04	<0.00001	87%
	Functional training program	13	3.38	2.47, 4.30	<0.00001	95%
Time (min)	Under 60 min	8	3.64	2.45, 4.83	<0.00001	95%
	60 min	9	2.17	1.67, 2.67	<0.00001	79%
	More than 60 min	3	4.25	0.71, 7.79	0.02	98%
Frequency (time/week)	Under 3 times/week	5	3.65	1.74, 5.56	0.0002	96%
	3 times/week	12	3.13	2.39, 3.87	<0.00001	94%
	More than 3 times/week	4	2.15	0.94, 3.35	0.0005	85%
Period (week)	6 weeks	2	3.09	−1.92, 8.11	0.23	98%
	8 weeks	5	2.71	1.45, 3.96	<0.0001	86%
	12 weeks	12	2.80	2.24, 3.35	<0.00001	92%

## Data Availability

The data included in this study are available and can be accessed by contacting the corresponding author.

## Supplementary Materials

The following are available online at https://www.mdpi.com/article/10.3390/ijerph19159353/s1, Table S1: Search history.
