# Supplementary material for: Effects of Exercise-Based Interventions on Functional Movement Capability in Untrained Populations: A Systematic Review and Meta-Analysis
Source: Int J Environ Res Public Health. 2022 Jul 30;19(15):9353. doi: 10.3390/ijerph19159353 (PMC9368594; doi:10.3390/ijerph19159353)
Supplement: Supplementary file 1 [file ijerph-19-09353-s001.zip › ijerph-1828668-supplementary.pdf]

Table S1: Search history.

Databases	Search history
PubMed	(((functional movement screen[Title/Abstract]) OR (FMS[Title/Abstract])) OR (functional movement screen*[Title/Abstract])) AND (((((functional movement patterns[Title/Abstract]) OR (movement quality[Title/Abstract])) OR (injury risk[Title/Abstract])) OR (injury prediction[Title/Abstract])) OR (injury prevention[Title/Abstract])) OR (injur*[Title/Abstract])) AND((((exercise[Title/Abstract]) OR (physical activity[Title/Abstract])) OR (functional training[Title/Abstract])) OR (functional strength training[Title/Abstract])) OR (movement training[Title/Abstract])
Embase	#4 #1 AND #2 AND #3<br>#3 'exercise' OR 'physical activity' OR 'functional training' OR 'functional strength training' OR 'movement training':ab,ti<br>#2 'functional movement patterns' OR 'movement quality' OR 'injury risk' OR 'injury prediction' OR 'injury prevention' OR injur*:ab,ti<br>#1 'functional movement screen' OR 'FMS' OR 'functional movement screen*':ab,ti
Scopus	TITLE-ABS ("functional movement screen" OR fms OR "funcitonal movement screen*") AND TITLE-ABS ("functional movement patterns" OR "movement quality" OR "risk injury" OR "injury prediction" OR "injury prevention" OR injur*) AND TITLE-ABS (exercise OR "physical activity" OR "functional training" OR "functional strength training" OR "movement training")
